# Impaired specific CD8 T cell response with aging is not due to decreased expression of CD90 on TCR transgenic T cells

**DOI:** 10.1186/1742-4933-10-36

**Published:** 2013-08-19

**Authors:** Jiu Jiang, Erin Fisher, Donna M Murasko

**Affiliations:** 1 Department of Biology, Drexel University, 3245 Chestnut Street, Philadelphia, PA 19104, USA

**Keywords:** CD90, Transgenic, T cells, Aged mice

## Abstract

**Background:**

CD90 (Thy-1) is a small glycoprotein that is particularly abundant on the surface of mouse thymocytes and peripheral T cells, and is often used as a marker in adoptive transfer experiments to distinguish donor and recipient T cells with different CD90 subtypes. We have performed adoptive transfer experiments with T cell receptor transgenic (TCR Tg) mice to study the impaired CD8 T cell response with aging.

**Findings:**

After stimulation with a CD8 T cell epitope, HA_518-524_, the response of TCR Tg CD8 T cells from aged mice was decreased compared to the response of TCR Tg T cells from young mice. CD90 expression was also substantially decreased on the TCR Tg CD8 T cells of aged mice. However, the responses of CD90^hi^ and CD90^low^ CD8 T cells of the aged mice were similar in both early activation and proliferation, demonstrating that the impaired Tg T cell response with aging is not associated with the altered CD90 expression on CD8 T cells.

**Conclusions:**

The impaired Tg CD8 T cell response in aged mice is not due to age-associated changes in CD90 expression on Tg CD8 T cells.

## Findings

A decrease in the CD8 T cell response to virus infection with aging has been consistently observed [1-3], however, the mechanisms are still largely unknown. Both intrinsic and extrinsic factors are considered to affect the CD8 T cell response with aging [4,5]. The development of new immunological techniques, including T cell receptor transgenic (TCR Tg) mice [6-8], specific MHC tetramer staining [9], and lymphocyte sorting with flow cytometry, have significantly accelerated research concerning T cell immunity with aging [2,3,10]. Using an adoptive transfer approach, we observed that the aged environment significantly inhibits both clonal expansion and IFN-γ production by specific Tg CD8 T cells of young mice during virus infection [3], and that the decreased response of the Tg CD8 T cells transferred into aged mice could be significantly enhanced when DCs of young mice were co-transferred [11]. These results indicate that alterations in the aged environment play an important role in the decreased specific CD8 T cell immunity to virus infection with aging.

While the impairment of the aged environment affects the T cell response with aging, the intrinsic changes of T cells also play a critical role [5,12,13]. Since the percentage of specific CD8 T cells in wt mice is very low [14], we chose a TCR-Tg mouse model (i.e., Thy-1.1^+^Clone-4, which recognizes H-2K^d^ hemagglutinin (HA)_518–526_ CD8 T cell epitope of influenza virus [8]) to examine whether aging has an effect on the intrinsic response of CD8 T cells. Although aged Clone-4 mice are not commercially available, we aged the mice in our animal facility.

**Figure 1:**
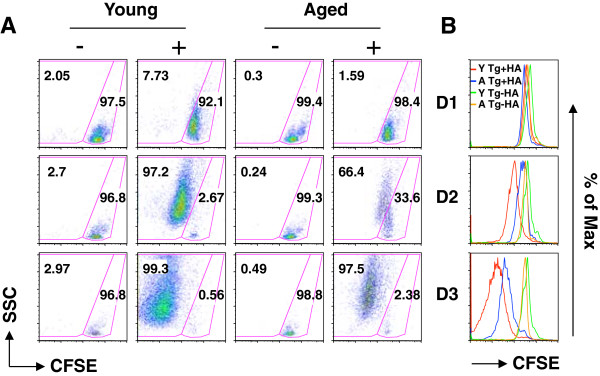
**Decreased response of T cells from aged Clone-4 mice after stimulation with HA**_**518-524**_**.** 1x10^5^ CFSE labeled splenocytes of young and aged Clone-4 mice (Thy-1.1^+^) were stimulated with or without 0.1 μM HA_518-524_ (+ or -). On Days 1, 2, and 3, cells were stained with anti-CD8 antibody. **(A)**. Each flow cytometric plot shows proliferation of CD8 T cells based on the loss of CFSE. **(B)**. Overlay of CFSE profiles of CD8 T cells from young and aged Clone-4 mice. Results are representative of three independent experiments with similar results.

Carboxyfluorescein succinimidyl ester (CFSE)-labeled splenocytes of young (2–3 month old) and aged (18–20 month old) Clone-4 mice were cultured with HA epitope. At different times post-stimulation (Days 1, 2, and 3), the proliferation of specific CD8 T cells was determined by flow cytometry based on the profile of CFSE, which is a vital fluorescent dye that is equally partitioned into daughter cells, allowing visualization of cell division [15]. The intensity, measured by mean fluorescence intensity (MFI) of CFSE, in cells continues to decrease as proliferation progresses. As shown in Figure 1A, little proliferation was observed in the CD8 T cells of either young or aged mice on Day 1. By Day 2, there was proliferation of both young and aged T cells, with a greater percentage of the Tg T cells of young mice demonstrating proliferation (young vs aged: 97% vs 66%). By Day 3, most of the T cells of both young and aged mice had proliferated (young vs aged: 99% vs 98%); however, more proliferation of the T cells of young compared to aged mice was observed based on lower MFI of CFSE (MFI: Young vs aged: 18 vs 72; Figure 1A &1B). These data demonstrate that the response of specific CD8 T cells of Clone-4 mice is both delayed and decreased with aging, similar to the response in wt mice infected with influenza virus [1,3].

**Figure 2:**
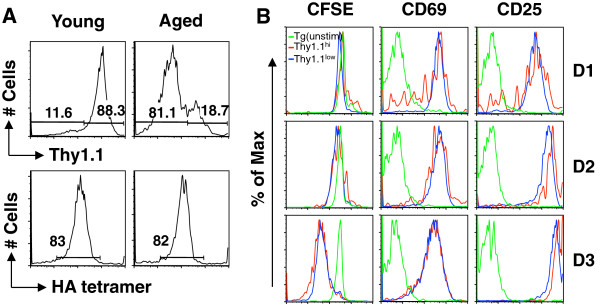
**Impact of altered CD90 expression with aging on the specific T cell response. (A)**. Splenocytes of naïve young and aged Clone-4 mice were stained with HA_518-524_ tetramer, anti-CD8, and Thy-1.1 antibodies and gated on CD8^+^ cells. **(B)**. 1x10^5^ CFSE labeled-splenocytes of aged Clone-4 mice were stimulated with or without HA_518-524_. On Days 1, 2, and 3, cells were stained with anti-CD8, Thy-1.1, CD69, and CD25 antibodies*.* The proliferation and early activation of CD90^hi^ and CD90^low^ cells are presented in each plot. Green line: Tg T cells/-HA; red line: Thy1.1^hi^/+HA; and blue line: Thy-1.1^low^/+HA. The experiment was performed three times with similar results.

**Figure 3:**
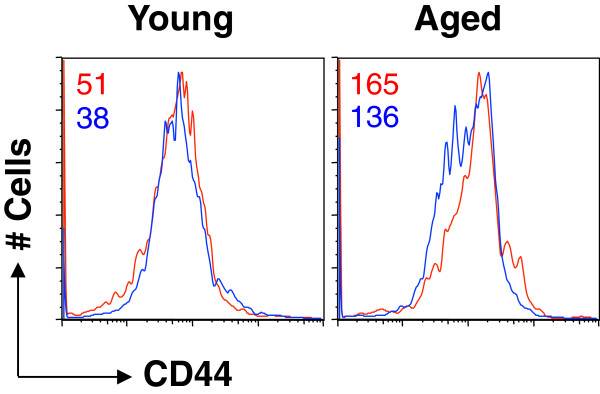
**CD44 expression in CD90**^**hi **^**and CD90**^**low **^**CD8 T cells of young and aged mice.** Splenocytes of young and aged Clone-4 mice were stained with anti-CD8, Thy1.1 and CD44 antibodies. The MFI level of CD44 on CD90^hi^ (red) and CD90^low^ (blue) CD8 T cells was examined; the number represents MFI of CD44 on CD90^hi^ and CD90^low^ cells. The experiment was performed three times with similar results.

**Figure 4:**
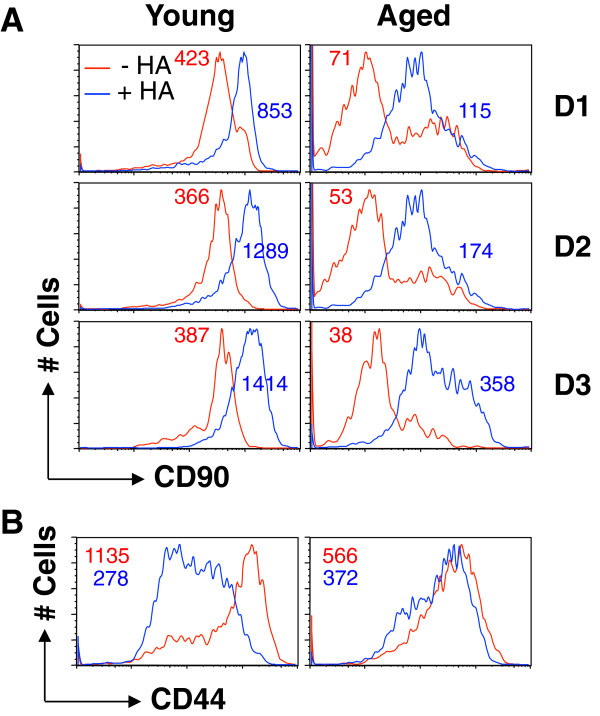
**Enhanced CD90 expression on CD8 T cells after stimulation *in vitro*.** Splenocytes of young and aged Clone-4 mice were cultured with or without HA_518-524_. **(A)**. On Days 1, 2, and 3, cells were stained with anti-CD8 and Thy-1.1 antibodies*.* The expression of CD90 on the CD8 T cells are presented in each plot. Blue line: +HA; red line: -HA. **(B)**. MFI of CD44 on CD90^hi^ (red) and CD90^low^ (blue) on Day 1 after stimulation. The number represents MFI of CD90 on the transgenic CD8 T cells. The experiment was performed three times with similar results.

CD90 (Thy-1), including subtypes Thy-1.1 and Thy-1.2, is a small glycoprotein that is particularly abundant on the surface of mouse thymocytes and peripheral T cells **[**[16,17]**]**. The CD90 molecule is often used in adoptive transfer experiments to distinguish donor and recipient T cells with different CD90 subtypes **[**[13,18]**]**. Since our previous adoptive transfer of Clone-4 Tg CD8 T cells from young mice (Thy-1.1) into aged recipients (Thy-1.2) utilized the Thy marker to differentiate donor and recipient CD8 T cells **[**[3]**]**, anti-Thy-1.1 is a standard antibody in our assessment of Tg T cells. To our surprise, CD90 expression on the CD8 T cells of the aged Clone-4 mice was substantially decreased (Figure 2A, top panel) compared to young Clone-4 mice. In contrast, the ability of Tg T cells of young and aged Clone-4 mice to bind HA tetramer was similar (Figure 2A, lower panel). CD90 has been reported to play a role in murine T cell activation since crosslinking CD90 molecules in the membrane raft results in the potent costimulation of T cells activated through the TCR **[**[17]**]**. Inhibition of T cell activation through down-regulation of TCR-CD3 expression can be mediated by an anti-CD90 antibody **[**[19]**]**. Furthermore, Thy-1 signaling promotes the *in vitro* generation of CTLs that kill target cells in a granule-dependent fashion **[**[20]**]**. To examine whether or not this age-associated change of CD90 expression contributes to the decreased T cell response with aging, we compared the responses of CD90^hi^ and CD90^low^ CD8 T cells of aged Tg mice. On Days 1–3 post-stimulation with HA peptide, the proliferation (CFSE profile) and early activation (upregulation of activation markers, CD69 and CD25) of the two populations were similar (Figure 2B). We also compared the expression of CD44 on transgenic CD8 T cells of young and aged mice. While the cells of young and aged mice displayed the expected difference in CD44 expression with cells of aged mice demonstrating higher expression (data not shown), there was no difference in CD44 expression between CD90^hi^ and CD90^low^ CD8 T cells of young or aged mice (Figure 3). After stimulation, the percentage of CD8 T cells demonstrating high levels of CD44 expression increased in both groups of mice (e.g., 24 h: Young: 56.5% to 84.8%, Aged: 73.8% to 86.6%). While the percentage of CD90^+^ CD8 T cells did not change with *in viro* stimulation (data not shown), the expression of CD90 on the CD8 T cells of both young and aged mice was increased as indicated with MFI (Figure 4A). Interestingly, while there was no difference in CD44 expression of CD90^low^ and CD90^hi^ cells in aged mice, CD90^hi^ cells of young demonstrated higher CD44 expression than CD90^low^ cells (Figure 4B). These results demonstrate that the age-associated changes in CD90 expression on Tg CD8 T cells do not contribute to the decreased T cell response with aging. It is also important when utilizing Thy-1 as a marker for donor cells in adoptive transfer experiments to be aware of a possible decrease of the glycoprotein on CD8 T cells of aged mice.

In summary, our findings demonstrate that: 1) CD90 expression on Tg CD8 T cells is diminished in aged mice, and 2) the impaired Tg CD8 T cell response of aged mice is not due to the age-associated changes in CD90 expression on Tg CD8 T cells. While it is known that both intrinsic and extrinsic factors contribute to the decreased Tg T cell response with aging and the specific mechanisms are still under investigation, our data indicate that the age-associated change in CD90 on T cells is not a major indicator or contributor to the age-associated decrease in antigen-induced T cell proliferation.

## Abbreviations

TCR: T cell receptor; CFSE: Carboxyfluorescein succinimidyl ester; Tg: Transgenic; HA: Hemagglutinin; MFI: Mean fluorescence intensity.

## Competing interests

The authors declare that they have no competing interests.

## Authors’ contributions

JJ and DMM designed and planned the research, and wrote the manuscript. JJ and EF performed experiments and analyzed data. All authors read and approved the final manuscript.
